# Bioassay Studies Support the Potential for Iatrogenic Transmission of Variant Creutzfeldt Jakob Disease through Dental Procedures

**DOI:** 10.1371/journal.pone.0049850

**Published:** 2012-11-30

**Authors:** Elizabeth Kirby, Joanne Dickinson, Matthew Vassey, Mike Dennis, Mark Cornwall, Neil McLeod, Andrew Smith, Philip D. Marsh, James T. Walker, J. Mark Sutton, Neil D. H. Raven

**Affiliations:** 1 Health Protection Agency - Porton Down, Salisbury, Wiltshire, United Kingdom; 2 College of Medical, Veterinary & Life Sciences, Glasgow Dental Hospital & School, University of Glasgow, Glasgow, United Kingdom; 3 Leeds Dental Institute, Leeds, West Yorkshire, United Kingdom; Nagasaki University Graduate School of Biomedical Sciences, Japan

## Abstract

**Background:**

Evidence is required to quantify the potential risks of transmission of variant Creutzfeldt Jakob (vCJD) through dental procedures. Studies, using animal models relevant to vCJD, were performed to address two questions. Firstly, whether oral tissues could become infectious following dietary exposure to BSE? Secondly, would a vCJD-contaminated dental instrument be able to transmit disease to another patient?

**Methods:**

BSE-301V was used as a clinically relevant model for vCJD. VM-mice were challenged by injection of infected brain homogenate into the small intestine (Q1) or by five minute contact between a deliberately-contaminated dental file and the gingival margin (Q2). Ten tissues were collected from groups of challenged mice at three or four weekly intervals, respectively. Each tissue was pooled, homogenised and bioassayed in indicator mice.

**Findings:**

Challenge via the small intestine gave a transmission rate of 100% (mean incubation 157±17 days). Infectivity was found in both dental pulp and the gingival margin within 3 weeks of challenge and was observed in all tissues tested within the oral cavity before the appearance of clinical symptoms. Following exposure to deliberately contaminated dental files, 97% of mice developed clinical disease (mean incubation 234±33 days).

**Interpretation:**

Infectivity was higher than expected, in a wider range of oral tissues, than was allowed for in previous risk assessments. Disease was transmitted following transient exposure of the gingiva to a contaminated dental file. These observations provide evidence that dental procedures could be a route of cross-infection for vCJD and support the enforcement of single-use for certain dental instruments.

## Introduction

vCJD remains a challenge for public health due to uncertain prevalence in the population and the possibility of cross-infection through medical procedures. The disease almost certainly emerged due to the consumption of bovine spongiform encephalopathy (BSE)-infected meat [1] but clinical cases have not reflected the widespread exposure of the UK population. The possibility of a self-sustaining and potentially amplifying “epidemic”, caused by the iatrogenic transmission of vCJD from pre-symptomatic cases and asymptomatic carriers to more genetically susceptible individuals, is a major concern.

The prevalence of the disease in the population is estimated at between *237* and 109 vCJD carriers per million of the UK population (95% confidence limits 49–692 per million [2] and 3–608 per million [3], respectively) All clinical cases of vCJD, to date, have been PRNP-129 Met homozygotes, but pre-/sub-clinical carriage has been identified in 2 valine homozygotes and a heterozygous patient [2] [4] [5]. Extended asymptomatic incubation periods in these genotypes have been suggested by transgenic animal studies [6] and also by studies on Kuru [7]. A recent study has identified a patient with atypical sporadic CJD and valine homozygous at PRNP codon 129 [8] which could represent the first case of clinical disease in this genogroup. Aside from blood transfusion [5], [9] there remains no evidence of iatrogenic vCJD transmission to date via any surgical route.

The potential transmission of vCJD by dental practice remain poorly defined. A risk assessment carried out by the Department of Health in 2004 (http://www.dh.gov.uk/en/Publicationsandstatistics/Publications/PublicationsPolicyAndGuidance/DH_4084662; last accessed 12^th^ November 2011), suggested a low level of risk, based on the assumption that there would be insignificant levels of infectivity except within the dental pulp and that only dental instruments which contacted this material posed any risk of cross infection. These assumptions are tested in this study. This risk assessment was revised in 2007 (http://www.dh.gov.uk/en/Publicationsandstatistics/Publications/PublicationsPolicyAndGuidance/DH_081170; last accessed 12^th^ November 2011), based on data which includes the preliminary outputs of this study.

Studies have described the presence of infectivity in hamsters following intraperitoneal challenge with 263 K scrapie [10], with 7.2 (gingiva) and 5.6 (dental pulp) log LD_50_ i.c. units (the dose capable of causing the death of 50% of challenged animals when injected intracranially into hamsters) per gram of tissue. The study also showed that scrapie could be transmitted through injection into the dental pulp. A recent study has shown infectivity in the root of the right caudal incisor tooth in an ME7-scrapie infected mouse following intracerebral challenge [11]. Collectively these studies suggest the potential for transmission of the disease via the oral cavity, but comprehensive data, particularly using prion strains more directly relevant to modelling vCJD in humans, remains lacking.

Bioassays using tissues from vCJD patients are underway (Sutton et al unpublished), but no disease-associated prion protein (PrP^Sc^) staining has been observed in any oral tissue from vCJD patients [12]. With very small number of samples involved and the absence of direct transmission from human tissue to animals in low titre vCJD tissues (<10^3^ ID/gram tissue; [13]), rodent-passaged TSE strains are essential to assess the relative levels of infectivity in different tissues, following exposure by different routes, as well as data on spread of disease.

The present study provides evidence on the potential risks of vCJD transmission by measuring relative levels of infectivity in oral tissues and assessing the potential for transmission through contact of a contaminated instrument with the gingival margin.

## Methods

### Primary challenge of VM mice via the small intestine

All studies were conducted under a project license approved by the UK Home Office. Prior to submission for approval the license was reviewed by the Microbiological Services Porton Ethical review committee and signed off by the Establishment Certificate Holder. Project license 30/2700 was granted by the UK Home Office under the Animals (Scientific Procedures) Act, 1986. A volume of 100 µl of a 2% (w/v) titred stock of BSE301V-infectious mouse brain homogenate (estimated titre 10^8.9^ infectious units per gram brain) [14] was injected into the lumen of the upper small intestine. Groups of 10 VM ((*Sinc^P7P7^* mice [15]. mice (8–10 weeks old) were anaesthetised by intraperitoneal injection of a mixture of Hypnorm (fentanyl/fluoanisone) and Hypnovel (midazolam) (Schering-Plough Animal Health, Welwyn Garden City, UK). With the animal in dorsal recumbency, a small incision was made in the skin of the upper abdomen, the upper loop of the jejunum just posterior to the duodenum was visualised and an injection made through the mesenteric membrane using a 1 ml syringe with a 30G needle. Groups of 10 mice were sacrificed at 3-weekly intervals (3 to 21 weeks) post-inoculation (p.i.) or on appearance of defined clinical symptoms at around 22–24 weeks [14].

### Primary challenge of VM mice via transient exposure of the gingival margin

Dental files were selected to perform the study, due to their relatively small size and ease of handling. Size “08” (21 mm) dental files were immersed in 10% brain homogenate and incubated for 30 minutes. Files were removed, and air dried at room temperature for 1 hour.

The mice were fully anaesthetized, as above, and the infected dental file was gently inserted into the mouth of the mouse in parallel with the right jawbone at the height of the gingival margin. It is highly likely that the far point of the file (up to a maximum of 1 mm will have entered the outer layer of the gingival epithelium (but not the area known as the gingival sulcular epithelium adjacent to the tooth socket). Due to the parallel placement, this penetration would have been at a very glancing angle to the tissue and the majority of the file was thus left lying in parallel contact with the gingiva along the length of the jaw (jaw about 6 to 7 mm length; contact region with file estimated at around 5 mm) for the designated 5 minute period, after which it was gently withdrawn. Due to the serrated nature of these files damage to the epithelium cannot be ruled out, but on no occasion was there any trauma or bleeding observed during or after this procedure so any damage to the epithelium will have been minimal.

The maximum load of infectivity on coated dental files was estimated. The dental files are manufactured as a morse taper with an end diameter of 0.08 mm. Assuming a 5 mm section was inserted into the mouth, the maximum diameter would be around 0.18 mm. Surface area of a plain wire would be approximately 2 mm^2^ (2π(r_av_)h+πr_end_^2^). The fluting is assumed to increase the area by no more than 5 fold (maximum surface area 10 mm^2^). Previous studies using a similar coating strategy have suggested retention of approximately 0.2 µg brain tissue per mm^2^ [15]. Based on a titre of 10^8.9^ ID_50_ per gram brain [14] the maximum load of infectivity on the dental file is estimated at 4×10^2.9^ ID_50_ per challenge).

Groups of 10 mice were sacrificed at 4-weekly intervals (1–6 months) post-inoculation (p.i.) or on appearance of defined clinical symptoms [14].

### Analysis of time-course samples

The whole brain (including the medulla oblongata), spleen, salivary gland, trigeminal ganglia, dental pulp, gingival margin, lingual muscle (front 2/3rds of the tongue), lingual tonsil (back 1/3 of tongue including tonsular tissue), salivary gland (submandibular) and saliva (following pilocarpine stimulation) were collected from mice at the different time points. The individual tissues from each time point were pooled and stored at −80°C prior to re-inoculation. Tissue homogenates were prepared at 20% (w/v) tissue in phosphate buffered saline using a Ribolyser (Fast prep 120A; Q-Biogene). As the weight of tissue from the dental pulp could not be measured this tissue was diluted to the minimum volume of homogenate required for re-inoculation. Ribolyser beads were washed with 100 µl PBS, which was used to dilute the homogenates to 10% (w/v) prior to inoculation.

**In vivo analysis:** The infectivity of the tissues was assessed by i.c. inoculation into the brains of VM mice. Groups of 6 VM mice (6–8 weeks old) were anaesthetised by intra-peritoneal injection with alfaxalone/alfadolone (Saffan, Schering-Plough Animal Health, Welwyn Garden City, UK) and inoculated intra-cranially with 20 µl of the 10% homogenate. Non-specific toxicity was observed in some groups and samples were diluted (to 1% or 0.1%) as required.

Mice were monitored for clinical symptoms and sacrificed by injection of barbiturate (pentabarbitone sodium) at a defined clinical end-point. Brains from indicator mice were removed and stored in formalin prior to histological assessment by Animal Health and Veterinary Laboratories Agency, Weybridge, UK.

**In vitro analysis:** Homogenates were analysed by Western blot essentially as described previously [14]. In brief, homogenates were digested with Proteinase-K at a final concentration of 5.37 µg/ml for 30 minutes at 60°C. The enzyme was inactivated by incubation with 5 mM APMSF (Sigma, Gillingham, UK) in Nu-Page™ gel loading buffer (Invitrogen, Paisley, UK) at 99°C for 10 minutes. Samples, together with the relevant controls, were run on 4–12% Bis Tris NuPage gels (Invitrogen, Paisley, UK) and transferred to nitrocellulose. The membrane was blocked in 5% skimmed milk powder in phosphate buffered saline containing 0.1% Tween 20 (PBS-T) for 30 minutes, washed in PBS-T and incubated with primary antibody 6H4 (Prionics, Schlieren, Switzerland) (at 1∶10,000 dilution) for 18 hrs at 4°C. The membrane was washed four times in PBS-T and bound antibody was detected with anti-mouse horse radish peroxidase (HRP)-conjugate (Sigma, Gillingham, UK); diluted 1∶1000). Signal was generated using West Dura reagent (Pierce, Cramlington, UK) and imaged using a Chemidoc image analyser (Pharmacia, Sandwich, UK). The Western blot method could not detect signal below a gel loading equivalent to a 0.1% brain homogenate (results not shown).

## Results

### Primary transmission of infectivity from the small intestine to simulate oral exposure to BSE

Mice were challenged via direct inoculation into the small intestine to avoid any chance of contamination of the oral tissues during the primary challenge. Disease transmission was observed in all animals, with a mean incubation period to a defined clinical endpoint of 157±17 days (Table 1A). Previous studies have shown that direct i.c. challenge with the same titre of infectious BSE-301V stock (estimated titre 10^8.9^ infectious units per gram brain [14]) reaches a clinical end-point in 120±8.5 days.

**Table 1:** 

Table 1A: Summary of primary challenge data for different transmission routes		
Challenge Route	Attack rate (number of animals succumbing to disease / number of animal challenged (% attack rate))	Mean incubation / days post infection ± standard deviation
Small intestine challenge	46/46 (100%)	157 ± 17*
Gingival margin challenge	68/70 (97.1%)	233 ± 33.4^¶^

Table 1B: Separate analysis of primary cull data from gingival challenge shows two populations		
Challenge Route	Attack rate (number of animals succumbing to disease / number of animal challenged (% attack rate))	Mean incubation / days post infection ± standard deviation (range). Data for TSE positive animals only.
Gingival margin; Early terminal only	11/11 (100)	166 ± 18 (140-188)
Gingival margin; Late terminal only	57/59 (96.6%)	247 ± 14 (211-275)

* range 131–230 days, median 153 days; 1 mouse died without clinical BSE symptoms at 422 days post-challenge, with no histological confirmation of BSE and was excluded from the calculation (otherwise 178±67 days). Outlier at 230 days; otherwise 156±14 range 131–192.

### Analysis of relative levels of infectivity in oral tissues following simulated oral exposure

The levels of infectivity in different oral and control tissues were assessed by re-inoculation of 10% (w/v) tissue homogenate, intracranially into VM mice. The mean incubation period was compared to a titration series generated from BSE-301V terminal brain material as reported previously [14]. It is assumed in this study that serial dilution of infectivity would be unaffected by the tissue type and as such the incubation period can be used as an indication of the relative titre in the different tissues. In all cases shorter incubation to clinical symptoms is indicative of higher titre.

The study aimed to demonstrate the relative maximum levels of infectivity in different oral tissues following simulated food-borne exposure to BSE contamination. All tissues/fluids at the terminal stage of disease showed the presence of infectivity (Table 2). In all tissues except for the lingual tonsil, terminal tissues showed the maximal levels of infectivity recorded for that tissue. Incubation periods ranged from 118 days (±0 days, 2/2 animals infected) for brain tissue through to 213 days (±33 days, 4/5 animals infected) for lingual muscle tissue. In the case of lingual tonsil, the shortest incubation period (197±26 days) and highest attack rate (5/5) was reached by the 15 week time point. The lingual tonsil material from terminal animals showed lower levels of infectivity with only a single animal (1/6) succumbing to disease with an incubation of 222 days.

**Table 2:** Average incubation periods for VM mice challenged with tissues taken following small intestine challenge.

*Weeks*	*Brain*	*Brain 0.1%*	*Brain 0.1%*	*Spleen*	*Spleen repeat*	*Spleen: 1%*	*Spleen: 0.1%*	*Saliva*	*Gingival margin*	*Lingual muscle*	*Dental pulp*	*Trigeminal ganglion*	*Salivary gland*	*Alveolar bone*	*Alveolar bone repeat*	*Lingual tonsil*	*Lingual tonsil + synulox*
***3***	***233*** * N/A 1/3*			***129*** *±2 4/4*	***134*** *±0 5/5*			*0/6*	***273*** * N/A 1/5*	*0/4*	***269*** * N/A 1/6*	***168*** *±0 2/3*	***184*** *±22 5/5*		***230*** *±25 2/6*	*0/4*	***380*** * N/A 1/5*
***6***	***197*** *±26 5/5*			***142*** *±13 6/6*	***151*** *±23 2/2*			*0/6*	***243*** *±30 4/5*	***328*** *±193 3/6*	***192*** *±0 4/6*	***551*** * N/A 1/6*	***158*** *±4 5/5*		***257*** *±18 2/6*	*0/5*	***424*** *±73 2/6*
***9***	***147*** *±4 5/5*				***132*** *±9 6/6*	***170*** *±47 3/3*	***199*** *±105 4/5*	*0/6*	***284*** *±127 5/6*	*0/6*	***346*** *±90 3/5*	***249*** *±69 4/5*	***141*** *±3 6/6*		***231*** *±33 2/6*	*0/4*	***238*** *±18 2/6*
***12***	***118*** *±0 2/2*			***130*** *±0 4/4*	***130*** *±5 6/6*			*0/6*	***184*** *±16 5/6*	***344*** *±101 3/4*	***148*** *±0 6/6*	***140*** *±5 6/6*	***139*** *±7 6/6*		***192*** *±11 6/6*	***215*** *±39 5/5*	***190*** *±24 4/5*
***15***	***118*** *±0 6/6*	***135*** *±4 6/6*		***153*** *±15 4/5*	***178*** *±49 6/6*			*0/6*	***188*** *±28 3/6*	***204*** *±24 4/5*	***237*** *±40 5/6*	***120*** *±0 6/6*	***140*** *±0 6/6*		***172*** *±7 6/6*		***197*** *±26 5/5*
***18***		***118*** *±0 4/4*		***130*** *±0 3/3*				*0/5*	***153*** *±5 6/6*	***182*** *±21 4/5*	***156*** *±0 2/5*	***115*** *±9 6/6*	***134*** *±9 5/6*		***167*** *±8 5/5*		***196*** *±12 5/5*
***21***			***185*** *±114 6/6*	***137*** *±6 6/6*				*0/6*	***157*** *±6 6/6*	***236*** *±72 5/6*	***186*** *±27 6/6*	***121*** *±6 5/5*	***150*** *±5 5/5*		***183*** *±6 6/6*		***377*** *±245 2/6*
***Term.***			***118*** *±0 2/2*	***134*** *±6 6/6*				***207*** *±44 4/6*	***152*** *±0 6/6*	***213*** *±33 4/5*	***160*** *±55 6/6*	***136*** *±17 4/4*	***143*** *±6 6/6*	***158*** *±11 4/4*	***185*** *±15 6/6*		***222*** * N/A 1/6*

The mean incubation period (Bold), standard deviation (italics) and attack rate (mice infected/mice challenged) are all shown.

The oral tissues most likely to be contacted during routine dental surgery, (gingival margin and dental pulp), gave mean incubation periods of 152 days (±0 days, 6/6 animals challenged) and 160 days (±55 days, 6/6 animals challenged), respectively. To provide a comparison of the relative levels of infectivity, diluted brain samples gave mean incubation periods of 141±11 day (∼1000 ID_50_/milligram), 157±18.5 (∼100 ID_50_/milligram) and 226±94 days (∼10 ID_50_/milligram) ([14]). This suggests that gingival margin has between 100 and 1000 ID_50_/milligram, whilst dental pulp has at least 10 to 100 ID_50_/milligram given that the homogenate was less than 10% (w/v).

Maximal levels of infectivity were observed in all time course tissues, other than saliva, ahead of the appearance of any clinical symptoms. Maximal levels were reached by week 3 (spleen), 9, (salivary gland), 12 (brain, dental pulp, lingual tonsil), 15 (trigeminal ganglia, lingual muscle, alveolar bone), 18 (gingival margin), respectively. Clinical symptoms appeared around week 22, with these animals collected as the terminally diseased group. Saliva from terminal animals was the only time point which showed infectivity for this sample (mean incubation 207±44 days; 4/6 animal diseased).

By the first time-point at 3 weeks post-challenge, infectivity was already detected in the brain, spleen, trigeminal ganglia, gingival margin, dental pulp, salivary gland, alveolar bone, and lingual tonsil (with synulox), but not in lingual muscle or saliva. Incubation periods ranged from 129 days (±2 days; attack rate 4/4 animals) for spleen to 273 days (1/5 animals) for gingival margin (Table 2). The incubation period in the spleen sample was already at the minimum level, corresponding to a maximum level of infectivity for this tissue. By contrast brain tissue showed a mean incubation period of 233 days with only 1 of 3 mice that survived challenge developing disease.

The brains of all indicator animals were examined by staining with haemotoxylin and eosin (H&E) and by immunohistochemistry. All brain sections showed identical patterns of staining to that observed previously for this model ([14], [15]).

**Figure 1:**
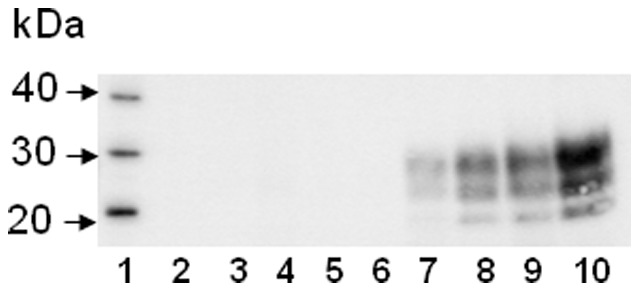
Detectable levels of PrP^Sc^ on Western blots do not correlate with the levels of infectivity. 10% brain homogenates from an uninfected brain (lane 2) time-course samples week 3, 6, 9, 12, 15, 18, 21 (lane 3–9), and the terminal sample (lane 10) were digested with proteinase K at 60°C for 10 minutes and assessed by Western blot. The observed signal does not correspond with the levels of infectivity found in corresponding bioassays for the week 12–21 post-exposure time-points.

Brain samples were also analysed by Western blot using antibody 6H4 following proteinase K digestion of the 10% homogenates (Figure 1). In contrast to the bioassay results, levels of detectable PrP^res^ varied significantly with the conventional triple glycoform banding pattern being observable in the 12 week brain samples only with extended exposure (results not shown) and increasing in the 15, 18 and 21 week samples to reach maximal levels only in the terminal group.

### Transmission of infectivity from the gingival margin following transient exposure

Dental files were used to assess whether short term contact was able to transmit infectivity via the gingival margin. The exposure was designed to mimic relatively atraumatic contact between a contaminated dental instrument and gingival epithelium (although limited abrasion of the gingival epithelium cannot be excluded – see materials and methods). The dental files were coated in 10% (w/v) brain homogenate to provide a worse case challenge via this route and in the absence of prior data on levels of infectivity in oral tissues.

**Figure 2:**
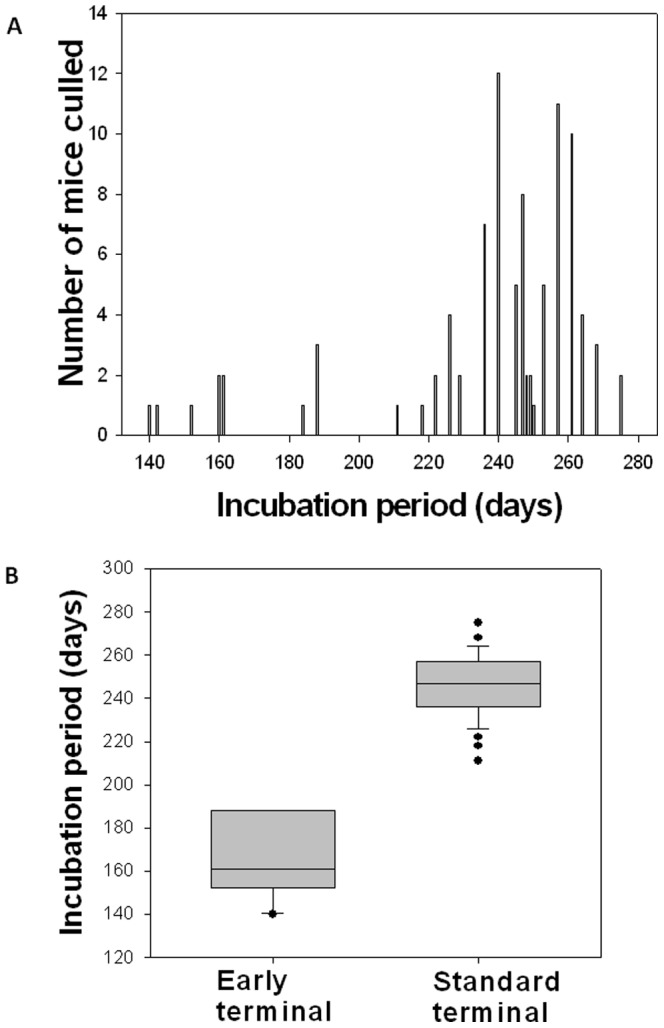
Comparison of the cull dates for the mice challenged via the gingival margin. Panel A; Frequency distribution plots show the presence of a normally distributed population with a mean incubation period of around 250 days plus a small number of animals with significantly shorter incubations ranging from 140–188 days. Panel B; when these two groups are compared they show distinct means and distribution and are considered as distinct populations (p<0.001).

Transmission via this challenge route was shown to be efficient with 97.1% (68/70) of challenged animals succumbing to disease. When the incubation period of individual animals was plotted (Figure 2A and B), two distinct incubation-period groups were identified (Student's T-test; p<0.001 [16], Sigmaplot version 10). The mean incubations for these two populations are shown separately in table 1B. The “early” terminal group had a mean incubation period of 166±18 days (n = 11; range 140–188) whilst the “standard” terminal group had a mean incubation period of 247±14 days (n = 57; range 211–275).

### Relative levels of infectivity in early and standard terminal groups, resulting from challenge via the gingival margin

The tissues from early and standard terminal groups were collected and processed as separate groups for re-inoculation into indicator mice (Table 3). The groups showed similar incubation periods in most tissues. Only alveolar bone (174±6 days, 6/6 animals challenged vs 160±5 days, 6/6 animals challenged) did not show overlapping standard deviations for early vs standard terminal groups, respectively. Comparisons were not made where there were less than 3 surviving animals in each challenged group (lingual muscle, saliva and gingival margin).

**Table 3:** Average incubation periods for indicator animals challenged with tissues taken following gingival margin challenge of VM mice.

*Months*	*Brain*	*Brain: 1%*	*Brain: 1%*	*Spleen*	*Saliva*	*Gingival margin*	*Lingual muscle*	*Dental pulp*	*Trigeminal ganglion*	*Salivary gland*	*Alveolar bone*	*Lingual tonsil + synulox*
***1***	*0/1*	*0/6*		***252*** *±11 2/6*	***353*** * N/A 1/5*	***314*** *±116 3/6*	***311*** *±36 4/5*	***255*** *±47 5/5*	*0/6*	***181*** *±13 6/6*	***262*** *±69 3/5*	***296*** * N/A 1/6*
***2***	***157*** *±12 3/4*			***163*** *±8 3/3*	*0/6*	*0/6*	***243*** *±63 5/6*	***244*** *±39 4/6*	***292*** * N/A 1/6*	***146*** *±8 4/4*	*0/6*	*0/6*
***3***	***147*** *±5 5/5*			***163*** *±11 5/6*	*0/6*	*0/3*	***249*** *±46 5/6*	***206*** *±30 5/6*	***317*** *±60 2/5*	***143*** *±5 5/5*	***281*** *±77 3/6*	*0/4*
***4***	***131*** *±2 4/4*			***162*** *±45 6/6*	*0/6*	***211*** *±24 3/5*	***226*** *±18 5/6*	***184*** *±7 6/6*	***214*** *±15 5/6*	***141*** *±0 6/6*	***200*** *±23 6/6*	***268*** *±88 3/6*
***5***	***124*** *±19 6/6*			***140*** *±4 6/6*	*0/5*	***189*** *±11 4/5*	***192*** *±10 6/6*	***228*** *±124 6/6*	***166*** *±17 6/6*	***136*** *±4 6/6*	***198*** *±34 3/6*	***205*** *±24 5/5*
***6***	***136*** *±7 3/3*			***141*** *±6 5/5*	*0/6*	***241*** *±32 4/4*	***206*** *±22 6/6*	***182*** *±13 6/6*	***289*** *±123 3/6*	***136*** *±3 6/6*	***297*** *±167 4/6*	***200*** *±5 2/6*
***Early terminal***	***120*** *±7 3/3*		***128*** *±4 6/6*	***150*** *±16 6/6*	***183*** * N/A 1/3*	***153*** *±0 2/2*	***155*** *±2 5/5*	***179*** *±19 6/6*	***159*** *±6 6/6*	***134*** *±3 5/5*	***174*** *±6 6/6*	***217*** *±64 5/5*
***Standard terminal***	***126*** *±5 6/6*			***134*** *±2 4/4*	***160*** * N/A 1/2*	***181*** *±0 2/2*	***175*** *±1 2/2*	***161*** *±10 4/5*	***160*** *±4 6/6*	***134*** *±3 5/5*	***160*** *±5 6/6*	***198*** *±43 3/5*

The mean incubation period (Bold), standard deviation (italics) and attack rate (mice infected/mice challenged) are shown for each tissue type taken through the time course. Based on the frequency distribution, two separate groups of terminal samples were taken and treated separately, termed early and standard terminal groups.

Again the brains of all indicator animals were examined by (H&E) staining and by immunohistochemistry. No differences were observed between any of the tissues from early versus standard terminal groups, and all results were consistent with previous studies ([14], [15]).

### Analysis of relative levels of infectivity in oral tissues following transient challenge via the gingival margin

Levels of infectivity were assessed as described above. Again, all tissues at the terminal stage of disease showed the presence of infectivity (Table 3). Incubation periods ranged from 126 days (+/−5 days, 6/6 animals challenged) for brain material to 198 days (+/−43 days, 3/5 animals challenged) for lingual tonsil, with all tissues showing maximal levels of infectivity in terminal animals. Saliva again showed infectivity only in terminally diseased animals (160 days, 1/2 animals challenged) and in a single animal at the earliest time point at extended incubation (353 days, 1/5 animals challenged) possibly due to persistence of the original inoculum.

Maximal levels of infectivity were again reached for all tissues (except for saliva) well ahead of the presentation of clinical symptoms by 4 months (brain and dental pulp) and 5 months (for all remaining tissues).

By the first time point in the time course, infectivity was detected in spleen, gingival margin, lingual muscle, dental pulp, salivary gland, lingual tonsil and alveolar bone, but not in brain, saliva or trigeminal ganglia. Incubation periods ranged from 181 days (+/−13 days, 6/6 animals challenged) for salivary gland to 314 days (+/−116 days, 3/6 animals challenged) for gingival margin. In several cases, notably in alveolar bone, lingual tonsil and gingival margin, infectivity was not observed in the 2 month time-point, nor in the 3 month time-point for gingival margin and lingual tonsil. This again may suggest localised persistence of the inoculum followed by clearance and later infiltration.

### Comparison of relative levels of infectivity between terminal groups challenged by the small intestine or gingival margin

The relative levels of infectivity were compared between terminally diseased animals from the two different challenge routes. Only the trigeminal ganglia (mean incubation 136+/−17 days, 4/4 for small intestine route vs 160+/−4 days, 6/6 for the gingival challenge route (standard terminal group) and 159+/−6 6/6 (early terminal group) did not show overlapping standard deviations. The lingual muscle samples were statistically different in the early terminal group from the gingival challenge route when compared to the small intestine challenge route (too few animals survived in the standard terminal group for valid comparisons to be made). Comparisons were not made where there were less than 3 surviving animals in each group (saliva and gingival margin in addition to the lingual muscle standard group).

At earlier time points accumulation of infectivity was proportionally slower in spleen and trigeminal ganglia than in the gingival challenge route. Spleen in particular showed much slower accumulation of maximal levels of infectivity, reached by week 3 in the small intestine challenge but not until month 5 in the gingival challenge group.

## Discussion

The principle aim of the study was to provide underpinning information regarding the potential risks of vCJD transmission by dental procedures, which would contribute to a revised dental risk assessment. The data provide an important insight into potential risks, albeit in a small animal model and using a worse-case approach.

The data presented here adds considerable information to the previous studies related to dental transmission [10]–[12]. The levels of infectivity observed in this study are lower than those seen in the Ingrosso study [10]. There are a number of potential reasons for this difference including; the challenge route used (intraperitoneal vs direct introduction to the small intestine), the higher titre of scrapie vs the BSE agent (typically 10^11^ ID_50_ per gram brain for 263 K Scrapie compared to ∼10^9^ ID_50_ for BSE-301V) and the different nature of the two prion agents themselves. As BSE-301V is derived from the same prion agent that caused vCJD in humans, it could be argued that the lower values are more representative of the levels of infectivity that might be encountered in dental patients. The absence of detectable disease-associated prion protein (PrP^Sc^) in human vCJD dental tissues [12] is not incompatible with the levels of infectivity observed in this study, given that the bioassay model is considered to be 100–1000 fold more sensitive than even the high sensitivity Western Blot model used in the cited study. Specifically, they identify that the levels of PrP^Res^ (ie the form of PrP that is resistant to digestion with Proteinase K; elsewhere termed PrP^Sc^) are at least 100-fold (dental, pulp and alveolar nerve) and 100-fold (gingival) less than that in matching grey-matter enriched brain tissue. The current study estimates infectivity in gingival margin to be between 100 to 1000 ID_50_/mg and dental pulp to be between 10–100 ID_50_/mg compared to the terminal brain containing approximately 1,000,000 ID_50_/mg (current study and [14]).

The re-infection studies carried out here are also more representative of the routine risks of disease transmission during dental procedures, than the highly invasive procedure used previously [10], where infectious brain material was injected directly into the pulp cavity.

The transmission of infectivity following direct inoculation into the small intestine proved to be highly efficient. This novel route of challenge probably accesses the same routes of infection that would be encountered after oral uptake of infectious material but without the significant reduction in titre (of the order of 2–3 log) expected on passage through the stomach. Whilst the approach will inevitably result in localised trauma at the incision site, the incubation period suggests that leakage into the peritoneum was not the primary route of infection as intraperitoneal challenge has resulted in animals reaching their clinical end-point at 196 days [17] with oral challenge at 245 days (unpublished; referenced in http://www.dh.gov.uk/prod_consum_dh/groups/dh_digitalassets/dh/en/documents/digitalasset/dh_081219.pdf (last accessed 12th November 2011)). Rapid accumulation of infectivity in the spleen, reaching maximal levels by the three week time-point, provides evidence of efficient infection through the small intestine.

The observed levels of infectivity, as estimated from incubation period, are higher than would have been expected in many tissues within the oral cavity. The two tissues most likely to be relevant to understanding the risks of iatrogenic dental transmission, the gingival margin and dental pulp, show levels of infectivity of between 100–1000 and at least 10–100 infectious doses (ID) per mg tissue, respectively (based on the titration series for brain material shown in [14]). The maximal levels of infectivity were reached well ahead of the presentation of clinical symptoms in the majority of tissues. This is likely to be similar in the human situation.

At the outset of the study, there was no indication in the literature that the two routes of infection would be as efficient as they proved to be. As such the study used a high challenge dose in order to be able to draw conclusions as to the spread of infection and accumulation of high levels of infectivity under worst-case conditions. Despite this, we do not believe that the use of a high challenge dose, distorts the key findings of the study. In the small intestine challenge experiments, the levels of infectivity in oral tissues are actually lower than the levels observed in the one limited but comparable study [10]. The accumulation of infectivity in the spleen is comparable to the rate seen in other peripheral challenges (intraperitoneal and oral) using the same model. The ability of the spleen to amplify infectivity from low-dose oral or peripheral challenge suggests that similar levels of infectivity would have been reached in the oral tissues even with a lower challenge. The different levels of infectivity and the different rate of accumulation of infectivity in different tissues also suggests that the model is not simply saturated with infectivity, but rather that it represents normal spread of infectivity from the intestine, potentially via both lymphoreticular and direct neuronal transmission.

The transient exposure of the gingival margin to infectivity dried onto dental files demonstrates the potential for iatrogenic transmission of infectivity through contaminated dental instrument contact within the oral cavity. The challenge was designed to be less invasive than previous oral inoculations [10] and gingival scarification [18]. Given the relatively atraumatic instrument contact, the efficiency of transmission was greater than expected with >97% of challenged animals succumbing to disease, with a total population mean of 233 days. The identification of two sub-populations within the culled animals on the basis of incubation period is intriguing. One of these populations could represent animals infected by ingestion of material following oral exposure. However, the use of a low challenge titre dried onto the file (estimated at around 4×10^2.9^ ID per file) and given the incubation period observed for much higher challenges via the oral route (245 days; see above), would suggest that ingestion is not the major infection route. The rapidly progressing (early) disease may be a result of localised trauma to the gingiva, providing more efficient spread of the disease, or may indicate that localised uptake has accessed different infection routes, perhaps mediated by neuronal (early terminal) and/or lymphatic (standard terminal) tissues, respectively. The relatively rich neurological innervations of the oral cavity and links with the trigeminal nucleus in the brain stem may contribute to this rapid route of spread. It might be expected that the two different routes of spread would show differences in the initial brain lesions, if the animals were analysed early in the infection process, before systemic spread of the prion agent throughout the brain. This was not investigated as part of the current study, as brains from primary infected animals were used for re-inoculation into indicator animals and as it would require *a priori* knowledge of which animals were infected by the different routes. Despite the significant differences in the incubation period of animals identified as early or standard terminal groups, widespread differences in the levels of infectivity in tissues were not observed on re-challenge. No difference in brain pathology were observed in indicator mice challenged with different tissue types from early and standard terminal groups, suggesting that no modification of the TSE strain had taken place during the primary challenge, irrespective of infection route.

The gingival challenge route is entirely novel and was designed to ask specifically whether infectivity could be transmitted via transient contact rather than direct inoculation [10]. To assess this, and given the very small amounts of inocula that are carried on the contaminated dental files, a high titre material was essential in order to test the feasibility of transmission. In terms of the validity of the model, the absence of infectivity at the 2 month time point for several tissues, including gingival margin, suggests that infection is not simply being generally disseminated through the oral cavity. Again this suggests that whilst the model is a worst-case the results are not incompatible with a natural infection from a contaminated instrument at lower titres.

Further discussion on relative levels of infectivity *in vivo* and PrP^Sc^-signal detectable *in vitro*, is provided in Supporting Information S1.

### Implications for public health

Currently there is no evidence for vCJD transmission through either surgery or dentistry. Transmission of vCJD by blood transfusion [5], [9] highlights that any procedure contacting nervous or lymphoid tissue must also be considered a risk given the wider tissue distribution of vCJD infectivity compared to sporadic CJD [19]–[21]. The highly efficient transmission of BSE strain 301 V infection through direct inoculation into the murine small intestine in this study raises similar concerns for vCJD transmission through endoscopic procedures in man.

The observations in the current study also provide theoretical grounds for concern in respect to dental procedures. The levels of infectivity observed in all oral tissues tested (most notably gingival margin with up to ∼1000 ID per mg) were higher than previously considered.

A further element of the study assessed residual protein contamination on a range of dental instruments after routine cleaning and disinfection in general dental practice in England [22]). The study showed a number of instrument types and cleaning procedures where the upper interquartile range for residual protein was in excess of 100 µg. This could equate to up to 100 ID per instrument in the case of gingival tissue. Autoclaving has been shown to achieve only a 3-log inactivation of various TSE agents [23] and an autoclave designed for the dental market has been tested recently and shown to provide only a 100-fold reduction in infectivity in the BSE301V/VM model used here (134°C, 18 minutes; Sutton et al unpublished). A dental instrument soiled with infectious gingival tissue and disinfected under this regimen would have an inadequate safety margin.

The gingival challenge was designed as a worse-case scenario in respect to the infectious load on a dental instrument, but to be of limited invasiveness. The procedure resulted in very high levels of transmission with short incubation periods indicating that a much lower titre challenge material would also have caused some transmission. Even if a relatively rare event, the large number of dental interventions taking place in a younger age profile population (c.f. surgical procedures) and a carrier population of unknown size means these risks are not negligible. This would seem to be at odds with the absence of any reported cases of clinical vCJD transmission linked to dental procedures. This might be explained by a number of factors, including difficulties in linking dental records to known vCJD patients [24], asymptomatic cases [5] and extended incubation periods for patients exposed by blood transfusion (up to 7.8 years; [25]). As a worse case study, the incubation periods described here would be expected to be the most rapid giving rise to prion-disease symptoms in this model, and as a novel low-dose, peripheral model of infection, the incubation periods might be expected to be considerably longer than those observed for blood transfusion cases. Given the difficulties in linking dental procedure case histories to vCJD, such cases may not yet be evident.

Preliminary data from this study have already been provided to the UK Department of Health as part of the revision of the dental risk assessment (http://www.dh.gov.uk/prod_consum_dh/groups/dh_digitalassets/dh/en/documents/digitalasset/dh_081217.pdf; accessed 12^th^ November 2011).

Additional control measures have been incorporated into guidance on decontamination in dental settings in England (http://www.dh.gov.uk/en/Publicationsandstatistics/Publications/PublicationsPolicyAndGuidance/DH_109363; accessed 12^th^ November 2011). The emphasis on standardised decontamination methods and single use instruments for difficult to clean devices appear sensible and proportionate given the experimental observations described and discussed here.

## Supporting Information

Supporting Information S1 Further discussion on relative levels of infectivity *in vivo* and PrP^Sc^-signal detectable *in vitro*.
(DOCX)
