# Supplementary material for: Bioassay Studies Support the Potential for Iatrogenic Transmission of Variant Creutzfeldt Jakob Disease through Dental Procedures
Source: PLoS One. 2012 Nov 30;7(11):e49850. doi: 10.1371/journal.pone.0049850 (PMC3511499; doi:10.1371/journal.pone.0049850)
Supplement: Supporting Information S1 — Further discussion on relative levels of infectivity in vivo and PrPSc-signal detectable in vitro. (DOCX) [file pone.0049850.s001.docx]

**Kirby et al;** Bioassay studies support the potential for iatrogenic transmission of variant Creutzfeldt Jakob disease through dental procedures

**S1 Supplemental discussion:**

***Relative levels of infectivity in vivo and PrP^Sc^-signal detectable in vitro.***

The presence of maximal levels of infectivity in the brain in mice at 12 weeks is a reminder of the long pre-symptomatic phase of TSE infection. The earliest clinical symptoms were evident at 18 weeks post-exposure with an average of 22 weeks. The 10-fold dilution data for the 15, 21 and terminal groups, although limited in nature, suggest that the levels of infectivity reach a maximal level and are maintained over this period, rather than continuing to accumulate in the brain. The observed differences in the level of abnormal prion protein, PrP^Sc^, in the same time-course samples indicates a marked separation between the level of infectivity and the detected level of its surrogate marker as observed in previous studies [S1,S2]. This may indicate that an equilibrium is reached for the most infectious form of the agent (e.g. the 14-28 unit prion protein oligomers [S3]) and that this remains unaltered despite the ongoing accumulation of Proteinase K-resistant aggregated PrP^res^. Alternatively, the overall level of infectivity may remain approximately constant since the capacity to act as individual nuclei of infection diminishes proportionately to the increasing extent of PrP^res^ aggregation. Neither hypothesis was tested in the current study.

Similar levels of infectivity were observed at the end of the two bioassay studies, with the exception of lingual muscle which was higher in mice challenged via the gingival challenge route. Comparing the levels of infectivity at the first point of each time course showed greater dissemination of infectivity within the oral cavity for the gingival challenged animals. This might have been expected with the tissues potentially retaining some of the initial inocula. This is supported by the subsequent loss of infectivity in three of the oral tissues by the second time-point, with gingival margin and lingual tonsil remaining non-infectious at the third monthly time-point. In contrast, organs which might be indicative of ingestion and systemic spread from the oral cavity remained less infectious for a greater proportion of the time-course. For example, the spleen showed only limited transmission and a longer incubation period following gingival challenge at the first time-point, with levels of infectivity not reaching those observed following challenge via the small intestine until the final terminal group. Similarly, neither trigeminal ganglia nor brain showed infectivity at the first monthly time-point and increased only gradually over time. The high levels of infectivity in the salivary gland at the first time-point and increasing through the incubation suggest that infectivity was concentrated and amplified in this organ rather than being disseminated from the descending nerves linked to the trigeminal ganglion, the latter showing lower levels of infectivity throughout the time-course.

***Supplemental references:***

S1. Sakaguchi S, Katamine S, Yamanouchi K, et al. (1993) Kinetics of infectivity are dissociated from PrP accumulation in salivary glands of Creutzfeldt-Jakob disease agent-inoculated mice J. Gen. Virol. 74: 2117-2123.

S2. Manson JC, Jamieson E, Baybutt H, et al. (1999) A single amino acid alteration (101L) introduced into murine PrP dramatically alters incubation time of transmissible spongiform encephalopathy EMBO. J. 18: 6855-6864.

S3. Silveira JR, Hughson AG, Caughey B. (2006) Fractionation of prion protein aggregates by asymmetrical flow field-flow fractionation Methods Enzymol. 412: 21-33.
